# Site‐Selective Fluorination of Bathocuproine Derivatives for Enhanced Performance and Stability in Perovskite Solar Cells

**DOI:** 10.1002/cssc.202501793

**Published:** 2025-10-24

**Authors:** Hong Nhan Tran, Doyeong Yeo, Dong‐Geon Kwun, Ramesh Kumar Chitumalla, Gyeong Cheon Choi, Joonkyung Jang, In Hwan Jung, Ji‐Youn Seo

**Affiliations:** ^1^ Department of Nano Fusion Technology Pusan National University Busan 46241 Republic of Korea; ^2^ BK21 FOUR Education and Research Division for Energy Convergence Technology Pusan National University Busan 46241 Republic of Korea; ^3^ Department of Organic and Nano Engineering and Human‐Tech Convergence Program Hanyang University 222 Wangsimni‐ro Seongdong‐gu Seoul 04763 Republic of Korea; ^4^ Humanoid Olfactory Display Innovation Research Center Pusan National University Busan 46241 Republic of Korea

**Keywords:** enhancement of buffer layers, modified bathocuproine, perovskite solar cells, thermal evaporation bathocuproine

## Abstract

Interface engineering is vital for optimizing charge transport, stability, and overall efficiency in perovskite solar cells. In this work, two novel fluorinated bathocuproine (BCP) derivatives, BCP‐m2F and BCP‐m4F, are introduced, featuring site‐selective monofluorination at the terminal phenyl rings. Compared to the previously reported BCP‐m1, which incorporates aryl substitution for improved planarity and charge transport, these new derivatives leverage fluorination to further tailor the electronic structure and interfacial behavior. The energy‐level modulation by fluorination plays only a minor role; however, fluorination significantly enhances device stability through stronger binding with C_60_ and a pronounced surface passivation effect. Experimentally, BCP‐m4F demonstrates superior film uniformity and conductivity compared to BCP‐m2F. Time‐resolved photoluminescence, *J*–*V* analysis, contact angle measurements, and damp heat stability test (ISOS‐D3) show improved charge extraction, reduced trap‐assisted recombination, increased hydrophobicity, and enhanced thermal and moisture stability, respectively. Notably, a device employing BCP‐m4F exhibits minimal open‐circuit voltage loss under low‐light conditions, highlighting its suitability for indoor or diffuse‐light applications. These findings underscore the potential of combining rational backbone design with targeted fluorination to achieve multifunctional interlayers that enhance performance and reliability in next‐generation perovskite solar cells.

## Graphical abstract

Two fluorinated bathocuproine (BCP) derivatives, BCP‐m2F and BCP‐m4F, are developed beyond the reported BCP and BCP‐m1. Fluorination enables tuning of electronic structure, dipole orientation, and interfacial properties. BCP‐m4F forms uniform, conductive, and water‐repelling films superior to BCP‐m2F. Devices with BCP‐m4F exhibit negligible open‐circuit voltage loss under low‐light conditions, confirming its strong potential for indoor solar cells.

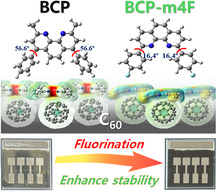

## 1 Introduction

**Figure 1:**
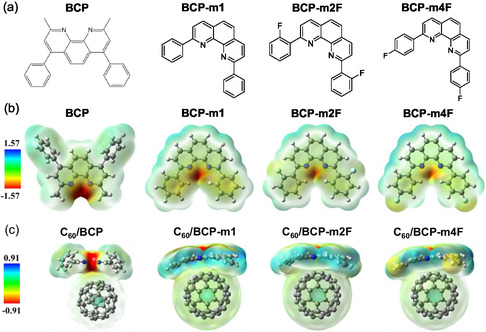
a) 2D chemical structures and b) electrostatic potential surfaces of BCP, BCP‐m1, BCP‐m2F, and BCP‐m4F. c) Electrostatic potential surfaces of C_60_/BCP, C_60_/BCP‐m1, C_60_/BCP‐m2F, and C_60_/BCP‐m4F complexes. Electrostatic potential values, shown on a color scale, are provided in electron volts (eV).

Perovskite solar cells (PSCs) have made remarkable strides in recent years, with single‐junction devices now surpassing 26% power conversion efficiency (PCE), drawing them closer to commercial viability. This progress has been driven not only by improvements in perovskite film quality and transport layers, but also by increasingly refined control of interfacial energetics and stability.^[^ ^1^ ^]^ One interface of particular interest is that between the electron transport layer (ETL) and the metal electrode, where Schottky barriers often limit carrier extraction and accelerate degradation.^[^ ^2^ ^]^ Bathocuproine (BCP), a small‐molecule buffer material, has thus become widely used in inverted (p–i–n) PSCs owing to its suitable lowest unoccupied molecular orbital (LUMO) level, solution‐ and vacuum‐process compatibility, and relatively high electron mobility.^[^ ^3^, ^4^, ^5^ ^]^ Despite its utility, BCP suffers from several critical drawbacks, particularly its vulnerability to moisture and its limited structural rigidity, which can undermine interfacial integrity and long‐term device performance. In our previous study, we reported a class of structurally modified BCP derivatives (e.g., BCP‐m1) that addressed some of these shortcomings.^[^ ^6^ ^]^ By introducing aryl substituents at the 2,9‐positions of the phenanthroline core, we enhanced molecular planarity, protected the electron‐rich nitrogen centers, and significantly improved charge transport properties and stability. Devices incorporating BCP‐m1 achieved superior performance compared to those using pristine BCP, validating the potential of rational backbone modification in interfacial materials design. Building upon this foundation, we now explore the targeted fluorination of BCP derivatives to further enhance interfacial functionality.^[^ ^7^, ^8^ ^]^ Fluorination has emerged as a powerful strategy in organic and hybrid photovoltaics for tailoring molecular energy levels, increasing dipole moments, improving hydrophobicity, and passivating interfacial defects.^[^ ^9^, ^10^, ^11^, ^12^, ^13^, ^14^ ^]^ In this work, we report two new fluorinated BCP derivatives (e.g., BCP‐m2F and BCP‐m4F) featuring selective monofluorination at the ortho and para positions, respectively, of the terminal phenyl rings (see **Figure** **1**a). Density functional theory (DFT) calculations predict that these substitutions lower the LUMO level, increase molecular rigidity, and enhance C_60_ binding affinity, while improving compatibility with the adjacent ETL and cathode. The newly synthesized BCP‐m2F and BCP‐m4F molecules exhibit excellent compatibility with vacuum thermal deposition, forming smooth and uniform films under standard processing conditions. Notably, BCP‐m4F displays improved film uniformity compared to its nonfluorinated analog BCP‐m1, suggesting that the introduction of fluorine enhances both molecular packing and deposition behavior. Experimental results support the theoretical predictions. Time‐resolved photoluminescence (TRPL) measurements exhibit extended carrier lifetimes, indicating effective trap passivation. Light intensity‐dependent *J*–*V* analyses further confirm suppressed trap‐assisted recombination and more efficient charge extraction. While the improvements in photovoltaic parameters, namely open‐circuit voltage (*V*_oc_), short‐circuit current (*J*_sc_), and fill factor (FF), are relatively modest, they are consistent and reproducible across devices. Importantly, contact angle (CA) measurements reveal a noticeable increase in surface hydrophobicity for the fluorinated derivatives, validating the hypothesis that fluorine incorporation effectively mitigates moisture ingress, which is a critical limitation of pristine BCP. Additional spectroscopic and morphological investigations, including photoluminescence quenching, electrostatic force microscopy (EFM), and impedance spectroscopy (IS), demonstrate reduced interfacial recombination, enhanced charge transfer dynamics, and improved interfacial adhesion. Devices employing BCP‐m2F and BCP‐m4F as buffer layers exhibit improved FFs, enhanced power conversion efficiencies, and significantly greater ambient stability compared to those utilizing conventional BCP or its nonfluorinated analog BCP‐m1. Taken together, our findings demonstrate that selective fluorination, when coupled with rational molecular backbone design, offers a promising route to engineer high‐performance, stable interfacial layers for next‐generation perovskite photovoltaics. In addition, fluorination was found to contribute more significantly to enhancing stability than to improving efficiency, primarily due to the stronger binding with C_60_ and the pronounced surface passivation effect induced by fluorination. This work highlights the synergistic effect of combining structural and chemical tuning strategies to overcome long‐standing limitations of small‐molecule buffer materials.

## 2 Results and Discussion

### 2.1 Synthesis of Characterization of Vacuum‐Deposition Novel Buffer Molecules

In our previous study, we investigated structural modifications of BCP to enhance the device performance and long‐term stability.^[^ ^6^ ^]^ The modified BCPs with improved molecular planarity showed enhanced intermolecular interactions with C_60_, which led to reduced film roughness and more efficient charge transport. In this study, to further improve not only the hole‐blocking properties but also the intermolecular ordering, fluorinated novel buffer molecules were synthesized via terminal molecular engineering of a 1,10‐phenanthroline‐based structure. The substitution of fluorine suggests potential for reduced hole leakage at the interface of the conjugated molecules. Moreover, fluorine substitution improves not only the intermolecular interactions but also the chemical resistance of the molecule,^[^ ^15^ ^]^ enabling it to serve as a protective barrier against the diffusion of water and oxygen from the air, thus suppressing the degradation of the perovskite layer. In particular, fluorine substitution at the 2‐ and 4‐positions is chemically more reactive owing to their ortho and para orientations, and these positional differences can affect the formation of the molecular dipole moment as well as intermolecular interactions.^[^ ^15^ ^]^ Therefore, both BCP‐m2F and BCP‐m4F were designed in this work. As shown in Scheme S1, Supporting Information, BCP‐m2F and BCP‐m4F were synthesized via a straightforward one‐step Suzuki coupling reaction of 2,9‐dichloro‐1,10‐phenanthroline with (2‐fluorophenyl)boronic acid and (4‐fluorophenyl)boronic acid, respectively, in the presence of a palladium catalyst and a base. The final products were obtained as white solids with yields of 73% and 61%, respectively, and exhibited good solubility in common organic solvents, such as chloroform. In addition, the molecular weights of BCP‐m2F and BCP‐m4F were tailored to ≈368 g mol^−1^, closely matching that of the original BCP (360 g mol^−1^), making them highly compatible with existing vacuum deposition processes. The newly synthesized BCP‐m2F and BCP‐m4F possess structures similar to control BCP‐m1,^[^ ^6^ ^]^ but fluorine atoms are substituted at the 2‐ and 4‐positions of the terminal phenyl ring, respectively. The molecular structures of BCP‐m1, BCP‐m2F, and BCP‐m4F were confirmed by ^1^H nuclear magnetic resonance (NMR) and ^13^C NMR, with the spectra provided in Figure S1–S6, Supporting Information.

To evaluate the thermal stability of the modified BCPs, thermogravimetric analysis (TGA) was conducted under a nitrogen atmosphere, as shown in Figure S7, Supporting Information. The BCP‐m1, BCP‐m2F, and BCP‐m4F exhibited similar thermal degradation profiles, with 5% weight‐loss temperatures of 300, 292, and 303 °C, respectively. The slightly reduced thermal stability of BCP‐m2F is presumed to originate from molecular structure distortion. Due to the *ortho*‐fluorine substitution, BCP‐m2F exhibits a larger dihedral angle compared to BCP‐m4F, which weakens the chemical bonding. However, despite their relatively small molecular weight in the range of 360–368 g mol^−1^, they possess sufficient thermal robustness for application in PSCs. The UV–visible absorption spectra of the BCP‐m1, BCP‐m2F, and BCP‐m4F were measured in both solution and film states. As shown in Figure S8a, Supporting Information, the absorption maxima of BCP‐m, BCP‐m2F, and BCP‐m4F were observed at 268 and 309 nm, 260 and 303 nm, and 268 and 311 nm, respectively, in solution, and at 272 and 314 nm, 262 and 303 nm, and 269 and 313 nm, respectively, in film states. Although BCP‐m2F exhibited a slightly blueshifted absorption wavelength compared to BCP‐m1 and BCP‐m4F, the difference was not significant. All the synthesized molecules showed similar absorption characteristics, with a significantly increased π–π* transition absorption and well‐developed vibronic band absorptions in the film. This indicates that fluorination of the terminal groups of the synthesized ETL materials has little impact on the absorption spectra, and that all the molecules exhibit excellent intermolecular interactions in the film state. The detailed optical properties are summarized in Table S1, Supporting Information. The optical bandgaps (*E*_g_^opt^) of BCP‐m1, BCP‐m2F, and BCP‐m4F were estimated from the absorption onset in the film state and were calculated to be almost similar to ≈3.3 eV. The highest occupied molecular orbital (HOMO) and LUMO energy levels of the synthesized ETL materials were determined using cyclic voltammetry (CV), based on the onset potentials of oxidation (*E*_ox_) and reduction (*E*_re_), as shown in Figure S8b, Supporting Information. The measured *E*_ox_ values of BCP‐m1, BCP‐m2F, and BCP‐m4F were respectively 1.69, 1.81, and 1.64 V, which corresponded to their HOMO energy levels of −6.01, −6.13, and −5.96 eV. Interestingly, it was observed that the position of fluorination has a significant impact on the HOMO energy level. BCP‐m2F showed ≈0.1 eV deeper HOMO energy levels than the others. It is expected that the electron‐withdrawing inductive effect of fluorine at the ortho position is stronger than that at the para position. This is in good agreement with the DFT calculations. The *E*_re_ values of BCP‐m1, BCP‐m2F, and BCP‐m4F were −0.77, −0.79, and −0.78 V, respectively, resulting in LUMO energy levels of −3.55, −3.53, and −3.54 eV, respectively. In the CV measurement, all three materials exhibited similar LUMO values, attributable to the unique reduction characteristics of the phenanthroline core structure inherent to all three.

To better understand the molecular structure, DFT simulations of BCP‐m1, BCP‐m2F, and BCP‐m4F were performed using Gaussian 16. Figure S9, Supporting Information, illustrates that BCP‐m2F and BCP‐m4F possess near‐planar geometries, similar to BCP‐m1, evidenced by dihedral angles of 17.1°, 17.2°, and 16.4° for BCP‐m1, BCP‐m2F, and BCP‐m4F, respectively. From the optimized geometries, we calculated the dipole moments (Figure S10a–c, Supporting Information), and the significantly higher dipole moment of BCP‐m4F (4.6661 D) compared to BCP‐m1 (2.1863 D) indicates a substantial increase in molecular polarity upon fluorine substitution at the 4‐position, likely due to the strong electronegativity of fluorine creating a large charge separation. Conversely, the much lower dipole moment of BCP‐m2F (0.6085 D) suggests that the fluorine at the 2‐position leads to a more symmetrical electron distribution where bond dipoles partially cancel, resulting in a less polar molecule overall. The charge transport capacity of BCP‐m1, BCP‐m2F, and BCP‐m4F was investigated by calculating their electron reorganization energies (EREs), which were then compared to that of reference BCP (Figure S10d, Supporting Information). The calculated EREs for BCP‐m2F and BCP‐m4F are significantly lower (262.6 and 261.6 meV, respectively) than that of BCP (424.5 meV), implying a potential for enhanced charge carrier mobility in the fluorinated derivatives, as a lower ERE typically facilitates charge transport. The calculated molecular electrostatic potential (ESP) surfaces reveal that fluorine substitution leads to a significant withdrawal of electron density from the aromatic phenyl rings (see Figure 1b). This electron‐withdrawing effect of fluorine reduces the electron richness of the phenyl rings, thereby lowering the energy barrier for electron injection and transport, ultimately facilitating improved electron transport properties. Moreover, the dispersion‐corrected binding energy (BE) simulations were performed to assess the binding strength of the BCP‐m1, BCP‐m2F, and BCP‐m4F with the C_60_. The BEs calculated for the interaction between the buffer layer materials and C_60_ reveal distinct binding strengths. Notably, BCP‐m2F exhibits the strongest interaction with C_60_, evidenced by a BE of −16.89 kcal mol^−1^. In contrast, BCP‐m1 and BCP‐m4F demonstrate slightly weaker, yet comparable, interactions with C_60_, with BEs −16.63 and −16.65 kcal mol^−1^, respectively. We summarized the theoretically evaluated data for the buffer materials in **Table** **1**. We evaluated the molecular geometries, electrochemical properties, and charge transport properties.

**Table 1:** Calculated structural and electronic properties of the BCP‐m1, BCP‐m2F, and BCP‐m4F buffer molecules, obtained at the B3LYP/6‐31 G(d,p) level of theory. The table includes the dipole moment, the dihedral angle between the phenanthroline and phenyl units, HOMO/LUMO energies, binding energy (BE) with C_60_, and electron reorganization energy (ERE).

Buffer molecule	Dipole moment [D]	Dihedral angle [^o^]	HOMO [eV]	LUMO [eV]	BE with C_60_ [kcal mol^−1^]	ERE [meV]
BCP‐m1	2.1863	17.1	−5.97	−2.41	−16.63	255.8
BCP‐m2F	0.6085	17.2	−6.10	−2.52	−16.89	262.6
BCP‐m4F	4.6661	16.4	−6.05	−2.53	−16.65	261.6

### 2.2 Morphology and Electrical Properties

**Figure 2:**
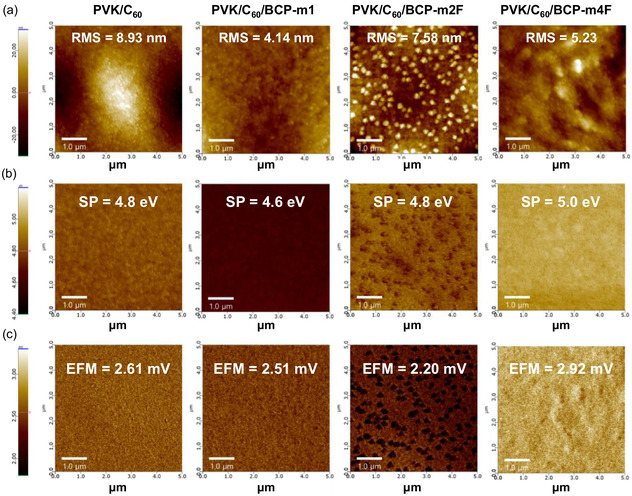
a) AFM, b) KFPM, and c) EFM surface electrostatic force images of C_60_, BCP‐m1, BCP‐m2F, and BCP‐m4F.

The morphology and surface potential (SP) of BCP on C_60_ were investigated by AFM (see **Figure** **2**a,b). Interestingly, BCP‐m1 and BCP‐m4F improve the surface roughness of the C_60_ film, reducing it from 8.9 to 4.14 nm and 5.23 nm, respectively. Such smoothing effects are advantageous for achieving uniform electrode deposition, minimizing contact resistance, and enhancing device stability. Notably, the BCP‐m2F surface reveals the presence of distinct aggregation domains, which may stem from its relatively low thermal stability, leading to clustering during thermal evaporation, as indicated by its lowest TGA decomposition onset temperature among the synthesized BCP variants. These morphological irregularities could adversely affect film homogeneity and charge transport, underscoring the importance of balancing molecular design with both thermal and interfacial properties. BCP‐m1 shows a lower SP than the reference C_60_, while BCP‐m2F and BCP‐m4F exhibit equal and higher SP, respectively. The enhancement in SP observed for the fluorinated derivatives is likely attributed to the strong electron‐withdrawing nature of fluorine atoms, which increases the local surface dipole moment. This electron‐withdrawing effect can localize electron density near the fluorine sites, thereby increasing the energy required to extract electrons from the C_60_/BCP‐F interface. Due to its relatively higher surface roughness compared to BCP‐m1, combined with a strong molecular dipole moment, BCP‐m4F exhibits the highest SP, likely resulting from the exposure of fluorine groups at the uneven surface.^[^ ^16^ ^]^ Additionally, the presence of fluorine‐containing groups at the surface may protect the underlying layers by repelling moisture, which could be beneficial for long‐term device stability. Furthermore, EFM was employed to investigate the surface electrostatic characteristics of BCP‐m1, BCP‐m2F, and BCP‐m4F. EFM, operating in dynamic noncontact mode, enables the detection of electrostatic interactions between a conductive AFM tip and the sample surface, thereby providing insights into the electrical behavior at the microscale. As shown in Figure 2c, the EFM phase images reveal distinct differences in electrostatic interactions, indicated by variations in color contrast. The BCP‐m2F sample exhibits a predominantly brown color, corresponding to the lowest electrostatic force (EF) observed among the three materials. In comparison, BCP‐m1 and BCP‐m4F display a yellow color, indicating stronger electrostatic interactions. The mean EF values, determined from line profile analyses (see Figure S11, Supporting Information), were 2.51 mV for BCP‐m1, 2.20 mV for BCP‐m2F, and 2.92 mV for BCP‐m4F. These EF values are attributed to the local conductivity at the sample surfaces. During EFM measurements, a DC bias was applied between the conductive cantilever tip and the sample, inducing localized charge accumulation beneath the tip. Materials with higher electrical conductivity facilitate stronger electrostatic interactions between the tip and the sample. In the case of BCP‐m2F, it locally disrupts surface uniformity and charge continuity due to the presence of aggregation clusters on the surface, which hinder charge transport near the interface. On the other hand, BCP‐m4F exhibits the highest EF value, indicating superior surface conductivity, which is consistent with the results obtained from EREs calculations.

The surface energy of BCP‐m derivatives was examined through water CA measurements (see Figure S12, Supporting Information). Materials with low surface energy are typically more hydrophobic and exhibit higher water CA, whereas those with high surface energy tend to be more hydrophilic. The unmodified BCP exhibited the lowest CA of 69.1°, indicating weak water resistance due to the exposure of the pyridine nitrogen atom. Upon repositioning the distorted phenyl group in BCP‐m1, the CA increased significantly to 80°, suggesting that the phenyl group effectively shields the nitrogen atom, thereby reducing surface polarity. Further modification by introducing a fluorine atom at the 4‐position of the phenyl ring (BCP‐m4F) led to an even higher CA of 84°, attributed to the strong hydrophobic nature of fluorine, which contributes to a lower surface energy. The high CA of BCP‐m4F supports the hypothesis regarding the surface exposure of fluorine atoms, as seen in Figure 2b. Conversely, BCP‐m2F showed the lowest CA among the fluorinated derivatives (73°), likely due to its greater surface roughness. Additionally, the fluorine atom in BCP‐m2F is located at the 2‐position of the phenyl ring (i.e., closer to the pyridine group), which may also hinder its ability to block interactions between the nitrogen atom and water. This is supported by the large dihedral angle observed for BCP‐m2F in Figure S9, Supporting Information, which suggests partial exposure of the nitrogen atom, thereby reducing the material's hydrophobic character. Among all samples, BCP‐m4F demonstrates the lowest surface energy, reinforcing its pronounced hydrophobicity and highlighting the critical role of fluorine placement in modulating surface interactions.

### 2.3 Device Characterization and Performance Analysis

Since BCP serves as the buffer layer between the ETL and the electrode, its thickness is critical due to its influence on the electron tunneling effect. Figure S13, Supporting Information summarizes the device efficiency of BCP‐m2F and BCP‐m4F as a function of thickness, with detailed photovoltaic performance parameters presented in Table S2, Supporting Information. By applying the buffer layer, energy losses at the ETL/electrode interface are mitigated, thereby enhancing the *V*_oc_. At very thin thickness (below 3 nm), the layer may become discontinuous or insufficient to form an effective tunneling barrier, leading to increased recombination or the formation of shunting pathways. Conversely, if the layer is too thick, it may impede efficient electron tunneling due to the exponential decay of tunneling probability with increasing distance. Therefore, precise control of the buffer layer thickness is essential to ensure effective charge extraction while minimizing series resistance. The observed optimal thickness of 4 nm suggests that this value provides adequate surface coverage while still allowing efficient electron tunneling from the ETL to the electrode.

**Figure 3:**
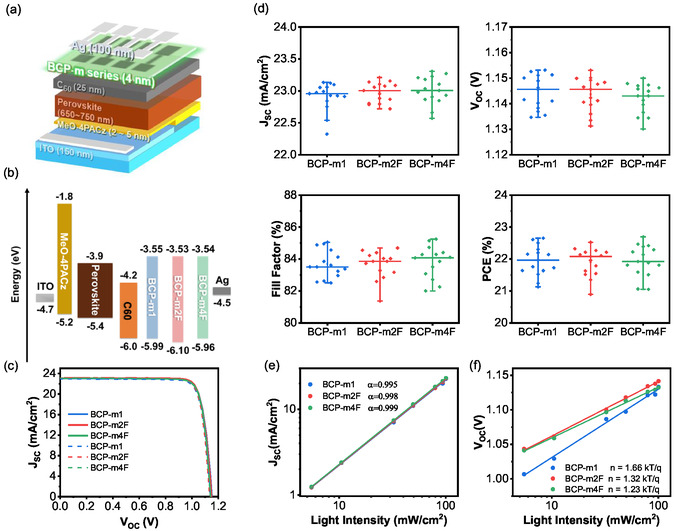
a) Device structure, b) energy level, c) *J*–*V* characteristics, and d) statistics graph of PSCs using BCP‐m1, BCP‐m2F, and BCP‐m4F. e) *J*_sc_ and f) *V*_oc_ of PSCs using BCP‐m1, BCP‐m2F, and BCP‐m4F as a function of light intensity.

To ensure a fair comparison, a uniform thickness of 4 nm was applied to all modified BCPs (BCP‐m), including BCP‐m1, BCP‐m2F, and BCP‐m4F. PSCs with the structure ITO/MeO‐4PACz/triple‐cation perovskite/C_60_/BCP‐m/Ag were fabricated, as illustrated in **Figure** **3**a. Based on CV measurements (see Table S1, Supporting Information), the corresponding energy band alignment is shown in Figure 3b. All BCP‐m derivatives exhibit a LUMO level of ≈3.55 eV, slightly deeper than that of pristine BCP (3.49 eV). This improved alignment with the LUMO of C_60_ facilitates more efficient electron injection from C_60_ to the Ag electrode, potentially enhancing device performance. Figure 3c and **Table** **2** show the *J*–*V* characteristics and device performance parameters for all BCP‐m, including the hysteresis test. Figure S14, Supporting Information shows the external quantum efficiency (EQE) spectra of the best device, and the EQE‐derived *J*_sc_ values match the *J*–*V* results within 5%, verifying the accuracy and reproducibility of the measurements. Despite differences in SP values, all BCP‐m derivatives exhibit similar performance characteristics with good hysteresis, especially BCP‐m4F (see Figure 3d and **Table** **3**). Light intensity‐dependent measurements were performed to investigate charge recombination at the C_60_/BCP‐m interface. Figure 3e,f shows the variation of *J*_sc_ and *V*_oc_ as a function of light intensity for PSCs incorporating different BCP‐m layers, with detailed *J*–*V* characteristics in Figure S15, Supporting Information. The *J*_sc_ follows a power‐law relationship with light intensity (*I*), expressed as $J_{\text{SC}}\;\propto I^{\alpha }$ where α is extracted from the linear fitting of ln(*J*_sc_)‐ln(*I*) plot.^[^ ^17^, ^18^ ^]^ In an ideal case under short‐circuit conditions, all photogenerated carriers are efficiently collected without bimolecular recombination, resulting in an α value of 1.^[^ ^19^, ^20^, ^21^ ^]^ On the other hand, the *V*_oc_ shows the linear relationship with the logarithm of the light intensity (ln *I*), which can be represented by equation $V_{\text{OC}}\propto n\left(\text{kT/q}\right)\text{ln}\left(I\right)$, where k is the Boltzmann constant, T is the absolute temperature, q is the elementary charge, and the ideality factor n is determined from the linear fitting of *V*_oc_‐ln(*I*) plot. An ideal factor *n* value (i.e, above one) typically indicates trap‐assisted recombination.^[^ ^22^, ^23^, ^24^ ^]^ Among the samples, BCP‐m1 exhibits the highest ideality factor of 1.66 kT q^−1^, compared to 1.32 kT q^−1^ for BCP‐m2F and 1.23 kT q^−1^ for BCP‐m4F. This increment of *n* value suggests a greater density of interfacial traps in BCP‐m1, a trend further supported by its lowest α value. The strong sensitivity of BCP‐m1 to low‐light conditions makes it less suitable for indoor PSC applications. The *V*_oc_ reduction under low‐light conditions can also be explained by the lower SP of the C_60_/BCP‐m1 interface compared to that of C_60_ alone, which creates an electron barrier. Under 1 sun illumination, the carrier density is high enough to bypass this barrier. However, under low‐light conditions, the carrier density is significantly reduced, making the barrier effect more pronounced. In contrast, BCP‐m4F demonstrates the lowest *n* and highest α value, indicating minimal trap‐assisted recombination and the lowest trap density among the BCP‐m series. As a result, BCP‐m4F is a promising candidate for indoor PSCs.

**Table 2:** Photovoltaic performance parameters of the PSCs using BCP‐m1, BCP‐m2F, and BCP‐m4F.

Description		JV *J*_sc_[mA cm^−2^]	*V*_oc_ [V]	FF [%]	Best PCE [%]	HI [%]
BCP‐m1	Forward	22.93	1.138	83.38	21.76	0.0194
	Reverse	23.06	1.151	83.61	22.19	
BCP‐m2F	Forward	23.14	1.133	83.72	21.96	0.0139
	Reverse	23.11	1.149	83.85	22.27	
BCP‐m4F	Forward	23.09	1.140	84.02	22.11	0.0014
	Reverse	23.08	1.141	83.85	22.08	

**Table 3:** Average photovoltaic performance parameters of the PSCs using BCP‐m1, BCP‐m2F, and BCP‐m4F.

Description	Average *J*_sc_[mA cm^−2^]	Average*V*_oc_ [V]	AverageFF [%]	AveragePCE [%]
BCP‐m1	22.91 (±0.16)	1.144 (±0.10)	83.69 (±0.71)	21.94 (±0.40)
BCP‐m2F	22.98 (±0.14)	1.144 (±0.10)	83.35 (±0.96)	21.90 (±0.36)
BCP‐m4F	23.01 (±0.17)	1.142 (±0.01)	83.53 (±1.11)	21.95 (±0.40)

For further verification, the IS was conducted to investigate recombination in PSCs using BCP‐m. Figure S16, Supporting Information shows the fitted resistance values for the devices measured under 1 sun illumination at various applied voltages. The inset displays the equivalent electrical circuit used for fitting.^[^ ^25^ ^]^ Note that the series resistance (*R*_S_) represents the contact resistance, and a constant phase element (CPE) was used to model capacitance in nonideal systems.^[^ ^26^, ^27^ ^]^ Across the entire voltage range, BCP‐m4F exhibited the highest recombination resistance (i.e., the lowest recombination rate), reaffirming its effectiveness in suppressing recombination. Finally, steady‐state PL and TRPL measurements were performed to examine the carrier lifetime (see Figure S17, Supporting Information). From the TRPL fitting, the average radiative recombination lifetime (τ_avg_) was extracted. BCP‐m4F exhibited the highest τ_avg_ of 25.02 ns, compared to 20.84 ns for BCP‐m1. In a system with a long carrier lifetime and fewer surface defects, nonradiative recombination, which dissipates energy as heat, is less common. Since BCP‐m4F shows fewer defects and a higher carrier lifetime than BCP‐m1, nonradiative recombination is reduced, thereby enhancing PL intensity through reutilization via interactions such as refraction, reflection, or scattering within thin films.^[^ ^28^ ^]^ Based on the IS, TRPL, and light‐intensity‐dependent analyses, it is confirmed that the novel BCP‐m4F possesses better electrical properties and lower *V*_oc_ light dependence compared to BCP‐m1.

**Figure 4:**
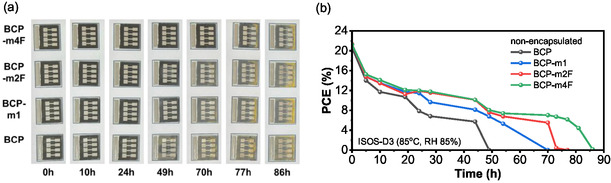
a) Photograph and b) summarized efficiency of PSCs using BCP, BCP‐m1, BCP‐m2F, and BCP‐m4F under the damp heat stability test (ISOS‐D3, 85 °C, RH 85%).

Finally, a damp heat test following the ISOS‐D3 protocol (85 °C, 85% relative humidity, RH) was conducted to further assess the environmental stability of the modified BCP. Note that the nonencapsulated devices were used to directly probe moisture‐induced degradation. The photograph of device degradation and the corresponding *J*–*V* characteristics are presented in **Figure** **4**a and S18, Supporting Information, respectively. All PSCs exhibited rapid performance loss within the first 10 h, followed by gradual degradation. Devices employing unmodified BCP fully degraded after 49 h, coinciding with the emergence of yellow discoloration due to PbI_2_ formation thus, indicating of perovskite decomposition. In contrast, devices with fluorinated BCP derivatives, particularly BCP‐m4F, demonstrated significantly improved stability, retaining performance up to 86 h (see Figure 4b). Moreover, maximum power point tracking measurements (ISOS‐L1, 1 sun, ≈45 °C, ambient air) of the encapsulated PSCs incorporating BCP derivatives were conducted (see Figure S19, Supporting Information). The enhancement of the initial PCE observed in these measurements can be attributed to the light‐soaking effect.^[^ ^29^ ^]^ Consistent with the damp heat test results, the fluorinated BCP derivatives exhibit higher stability than the nonfluorinated BCP. This enhancement is attributed to the hydrophobic nature of the fluorinated substituents, which suppresses moisture ingress and retard perovskite degradation.

## 3 Conclusion

In summary, we have rationally designed and synthesized two fluorinated BCP derivatives, BCP m2F and BCP‐m4F, demonstrating that site‐selective fluorination is an effective approach to modulate the electronic structure and interfacial properties of buffer layers in PSCs. Compared to the reported BCP‐m1, BCP‐m4F exhibits improved charge transport characteristics, reduced surface energy, and lower trap‐state density, as evidenced by CA measurements and light‐intensity dependent *J*–*V* characterization. Notably, BCP‐m4F demonstrates enhanced stability under damp‐heat conditions (ISOS‐D3, 85 °C, 85% RH) than BCP‐m1. Moreover, BCP‐m4F exhibits superior water repellency and thermal stability relative to BCP‐m2F, further contributing to its favorable interfacial behavior. Finally, the minimal *V*_oc_ loss under reduced illumination conditions underscores BCP‐m4F potential for efficient operation in low‐light environments.

## Conflict of Interest

The authors declare no conflict of interest.

## Supporting information

Supplementary Material

## Data Availability

The data that support the findings of this study are available from the corresponding author upon reasonable request.
