# Supplementary material for: Site‐Selective Fluorination of Bathocuproine Derivatives for Enhanced Performance and Stability in Perovskite Solar Cells
Source: ChemSusChem. 2025 Oct 24;18(24):e202501793. doi: 10.1002/cssc.202501793 (PMC12703419; doi:10.1002/cssc.202501793)
Supplement: Supplementary file 1 — Supplementary Material [file CSSC-18-e202501793-s001.pdf]

SUPPORTING INFORMATION

Site-Selective Fluorination of Bathocuproine Derivatives for Enhanced Performance and Stability in Perovskite Solar Cells

Hong Nhan Tran[1†], Doyeong Yeo[2†], Dong-Geon Kwun[1,3†], Ramesh Kumar Chitumalla[1], Gyeong-Cheon Choi[1,3], Joonkyung Jang [1], In Hwan Jung[2,] Ji-Youn Seo[2,3*]*

1 Department of Nano Fusion Technology, Pusan National University, Busan, 46241 Republic of Korea

2 Department of Organic and Nano Engineering and Human-Tech Convergence Program, Hanyang University, 222 Wangsimni-ro, Seongdong-gu, Seoul, 04763 Republic of Korea

3 Humanoid Olfactory Display Innovation Research Center, Pusan National University, Busan, 46241, Republic of Korea

E-mail: inhjung@hanyang.ac.kr; j-y.seo@pusan.ac.kr

Experimental Procedures

Materials: [1,1′-Bis(diphenylphosphino)ferrocene]dichloropalladium(II) was purchased from Sigma-Aldrich. 2-fluorophenylboronic acid and 4-fluorophenylboronic acid were purchased from TCI Chemical. 1,4-dioxane, CH_2Cl_2 and NaOH were purchased from DAEJUNG and 2,9-dichloro-1,10-phenanthroline was purchased from J's Science (South Korea).

Material synthesis

Synthesis of BCP-m2F:

2,9-dichloro-1,10-phenanthroline (500 mg, 2 mmol), 2-fluorophenylboronic acid, (616 mg, 4.4 mmol) and [1,1′-Bis(diphenylphosphino)ferrocene]dichloropalladium(II) (22 mg, 0.03 mmol) were dissolved in degassed 1,4-dioxane under nitrogen atmosphere and stirred until 80 °C. Then aqueous solution of NaOH (241 mg, 6 mmol, 1.5 ml) was added slowly to the reaction mixture and refluxed at 80 °C for 16h. The mixture was extracted with CH_2Cl_2 and washed several times with H_2O and brine. The combined organic layers were dried with anhydrous $MgSO_4$, filtered, and evaporated under reduced pressure. The crude product purified by column chromatography on silica gel using ethyl acetate/hexane (v/v, 1/6). **BCP-m2F** was obtained as a white solid (538 mg, 73%). ^{1}H NMR (600 MHz, $CDCl_3$) δ 8.54 (td, *J* = 7.9, 1.9 Hz, 1H), 8.32 (d, *J* = 8.4 Hz, 1H), 8.21 (dd, *J* = 8.4, 2.5 Hz, 1H), 7.84 (s, 1H), 7.49 – 7.43 (m, 1H), 7.39 (td, *J* = 7.5, 1.2 Hz, 1H), 7.21 (ddd, *J* = 11.6, 8.2, 1.3 Hz, 1H). ^{13}C NMR (101 MHz, $CDCl_3$) δ 161.16 (d, *J* = 249.9 Hz), 153.53 (d, *J* = 2.3 Hz), 146.32, 136.46, 132.20 (d, *J* = 2.9 Hz), 130.83 (d, *J* = 8.7 Hz), 127.90 (d, *J* = 11.1 Hz), 127.87, 124.78 (d, *J* = 3.4 Hz), 124.15 (d, *J* = 10.2 Hz), 116.13 (d, *J* = 23.1 Hz).

Synthesis of BCP-m4F:

2,9-dichloro-1,10-phenanthroline (500 mg, 2 mmol), 2-fluorophenylboronic acid (616 mg, 4.4 mmol) and [1,1′-Bis(diphenylphosphino)ferrocene]dichloropalladium(II) (22 mg, 0.03 mmol) were dissolved in 1,4-dioxane under nitrogen atmosphere and stirred until 80 °C. Then aqueous solution of NaOH (241 mg, 6 mmol, 1.5 ml) was added slowly to the reaction mixture and refluxed at 80 °C for 16h. The mixture was extracted with CH_2Cl_2 and washed several times with H_2O and brine. The combined organic layers were dried with anhydrous $MgSO_4$, filtered, and evaporated under reduced pressure. The crude product purified by column chromatography on silica gel using ethyl acetate/hexane (v/v, 1/4). **BCP-m4F** was obtained as a white solid (449 mg, 61%). ^{1}H NMR (400 MHz, DMSO) δ 7.80 – 7.61 (m, 3H), 7.55 (d, *J* = 8.4 Hz, 1H), 7.14 (s, 1H), 6.60 (t, *J* = 8.9 Hz, 2H). ^{13}C NMR (101 MHz, $CDCl_3$) δ 164.06 (d, *J* = 249.1 Hz), 155.89, 146.12, 137.17, 135.76 (d, *J* = 3.2 Hz), 129.61 (d, *J* = 8.5 Hz), 127.96,

126.15, 119.84, 115.92 (d, *J* = 21.6 Hz).

Materials characterizations:
^1H NMR spectra were recorded on VARIAN VNMRS 600MHz NMR spectroscopy and Bruker AVANCE III 400 spectroscopy, with tetramethylsilane as an internal reference. ^{13}C NMR spectra were recorded on Bruker AVANCE III 400 spectroscopy, with tetramethylsilane as an internal reference. The absorption spectra of organic materials were measured on a Jasco V-730 model UV-visible spectrophotometers. Cyclic voltammetry was performed on a WonATech ZIVE sp1 electrochemical analyzer with a three-electrode cell and Ag/Ag^+ reference electrode in a 0.1 mol L^{-1} Bu_4NBF_4 solution in acetonitrile at a scan rate of 100 mV s^{-1}. The working electrode was coated with the organic materials films by dipping them into their solutions in chloroform. The electrochemical potential of the materials was calculated with respect to the ionization potential of ferrocene/ferrocenium ion (Fc/Fc+) as an internal standard, -4.8 eV. The thermal stability of the materials was analyzed using a Discovery TGA550 Auto (TA Instruments) thermogravimetric analyzer. The measurements were carried out from 20 ^oC to 600 ^oC at heating rate of 10 ^oC min^{-1} under a nitrogen atmosphere.

Density functional theory (DFT) calculations:
Density functional theory (DFT) simulations were performed using the Gaussian 16 program[1] to elucidate the electronic structure of the BCP-m1, BCP-m2F, and BCP-m4F buffer materials and their interactions with C_{60}. The B3LYP functional[2-4] and the 6-31G(d,p) basis set were employed for all geometry optimizations. Frequency calculations were performed to confirm the nature of the stationary points as true minima. Electrostatic potential (ESP) surfaces were generated using GaussView[5] to analyze the charge distribution. Grimme's dispersion correction[6] (D3) was incorporated to estimate the interactions between the BCP molecules and C_{60} accurately. In addition, we employed the counterpoise method[7] to address the basis set superposition error.
The dispersion-corrected binding energy (BE) simulations were performed to assess the binding strength of the BCP-m1, BCP-m2F, and BCP-m4F with the C_{60}. Understanding the interaction strength between these buffer layer materials and C_{60}, which is vital for optimizing the performance of the PSC, relies on the analysis of their BEs. The BEs were determined using the formula: $\boldsymbol{BE = E_{Complex} - (E_{BCP} + E_{C60})}$ where, $E_{Complex}$, E_{BCP}, and E_{C60} are the energies of the BCP-C_{60} complex, BCP, and C_{60}, respectively. The BEs calculated for the interaction between the buffer layer materials and C_{60} reveal distinct binding strengths.

Device Fabrication:
The patterned indium tin oxide (ITO) substrates (1.1 mm-thick, 10 Ω/sq, AMG) were subjected to ultrasonication in 2% commercial detergent (Hellmanex) water solution, and isopropyl alcohol, in sequence, followed by drying in an oven at 100 °C for several hours. Once dried, the substrates were treated with UV for 15 min to increase their hydrophilicity. Subsequently, a 200 µl solution of Meo-4PACz in Methanol (0.36 mg/ml) was spin-coated on UV-ITO at 2000 rpm for 30s with a 2s acceleration and annealed at 100 ^oC for 10 minutes in a glovebox with a relative humidity below 10%. The preparation of the $Cs_{0.17}FA_{0.83}Pb(I_{0.88}Br_{0.12})_3$ perovskite precursor solution (1.45 M), FAI (187.0 mg), PbI2 (568.2 mg), FABr (13.6 mg), PbBr2 (79.8 mg), CsI (65.9 mg) were mixed in 1 mL anhydrous dimethylformamide/dimethylsulphoxide (9:1, v:v) solvent, and stirred at room temperature until completely dissolved. Oleylamine (0.004 M) was added into the perovskite precursor immediately before use. The solution was spin-coated at 3000 rpm for 10 s (with a 7-second acceleration) and then transferred to a vacuum chamber for a 15-second crystallization process, followed by annealing at 100 ^oC for 20 minutes on a hotplate. Following this, a 25 nm C_{60} layer was deposited by thermal evaporation at a rate of 0.2-0.4 Å/s. Subsequently, the modified BCP was

deposited at a rate of 0.1 Å/s. Finally, 100 nm of Ag was deposited by thermal evaporation at a rate of 0.8 Å/s.

Device characterizations:

Current density–voltage (*J*–*V*) characteristics were measured under AM 1.5G light (100 mW cm^{-2}) using the Xenon Arc lamp of an Oriel 69920 Sol3A class AAA solar simulator and the electrical signal from the cells was recorded in the forward direction from -0.1 V to +1.2 V using a Keithley 4200 source meter. Light intensity was calibrated using a standard Si solar cell. The active area defined by a metal mask aperture is 0.09 cm^2. External quantum efficiency (EQE) was characterized by an integrated system (PEC-S20) with a wavelength range of 300-850 nm. The non-encapsulated PSCs were placed in an environmental test chamber (JEIO TECH) at 85% humidity and a temperature of 85 ^{o}C. The non-encapsulated PSCs were illuminated under 1sun condition from white light-emitting-diode (LED) lamp in the ambient air without any cooling system, and biased at the maximum power point (MPP) voltage, and the power output was tracked by Arkeo multichannel (Cicci Research). SEM images were taken on a scanning electron microscope (Carl Zeiss Gemini500). The absorbance spectroscopy of the device was measured by the ultraviolet-visible and near-infrared (V-770 UV-Visible/NIR) spectrophotometer. The steady-state photoluminescence (PL) spectra and time-resolution photoluminescence (TRPL) were tested by Edinburgh FS5 Spectrofluorometer with the excitation light wavelength of 460 nm. Surface morphologies, Electrostatic distribution and Work functions were analyzed by NX20 AFM (Park Systems, Korea). NSCTAu tip was utilized during measurement. All the processes were conducted by Non-contact mode. The contact angle was measured using Smartdrop, and the surface energy was calculated with the measurement software. EIS analysis was measured using a Potentiostat
SP-300 (Bio-Logic SAS, France). To Impedance analysis, from 1 MHz to 1Hz range of frequency is set. From high frequency (1 MHz) to low frequency (1 Hz), interfacial contact resistance and recombination resistance characteristics can be measured. Especially in lower frequency, because electronic carrier densities can be affected by bulk electric field modulation with phase delay, recombination resistance can be analyzed.[8-10]

[redacted]

[redacted] SAS, France). To Impedance analysis, from 1 MHz to 1Hz range of frequency [redacted] equency (1 MHz) to low frequency (1 Hz), interfacial contact resistance and [redacted] tance characteristics can be measured. Especially in lower frequency, because [redacted] [illegible]

Scheme S1. Synthetic routes of BCP-m2F and BCP-m4F.

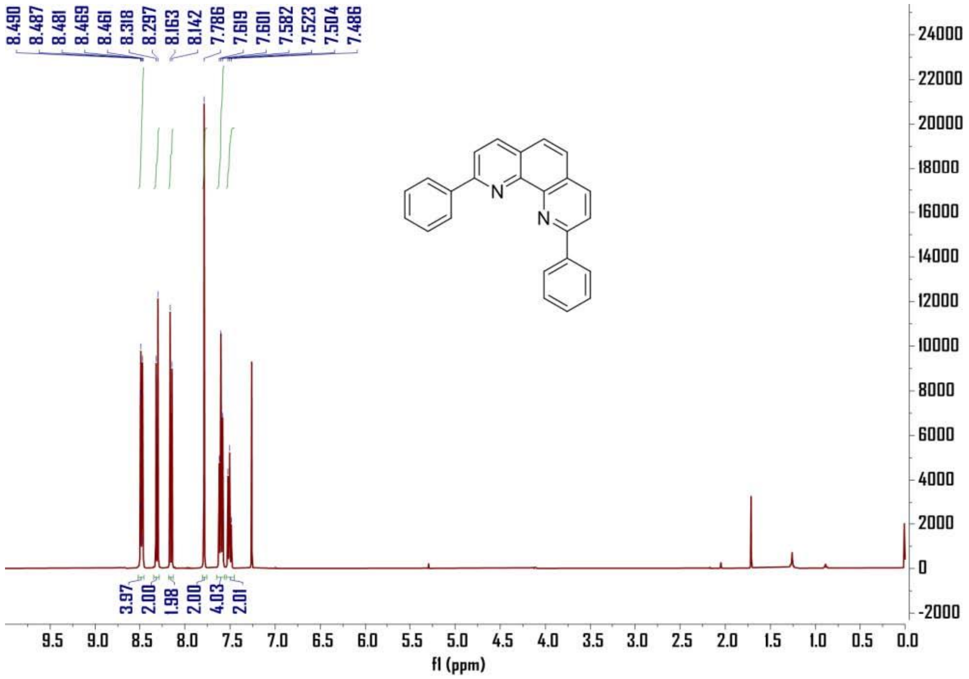

Figure S1. 1H NMR of BCP-m1.

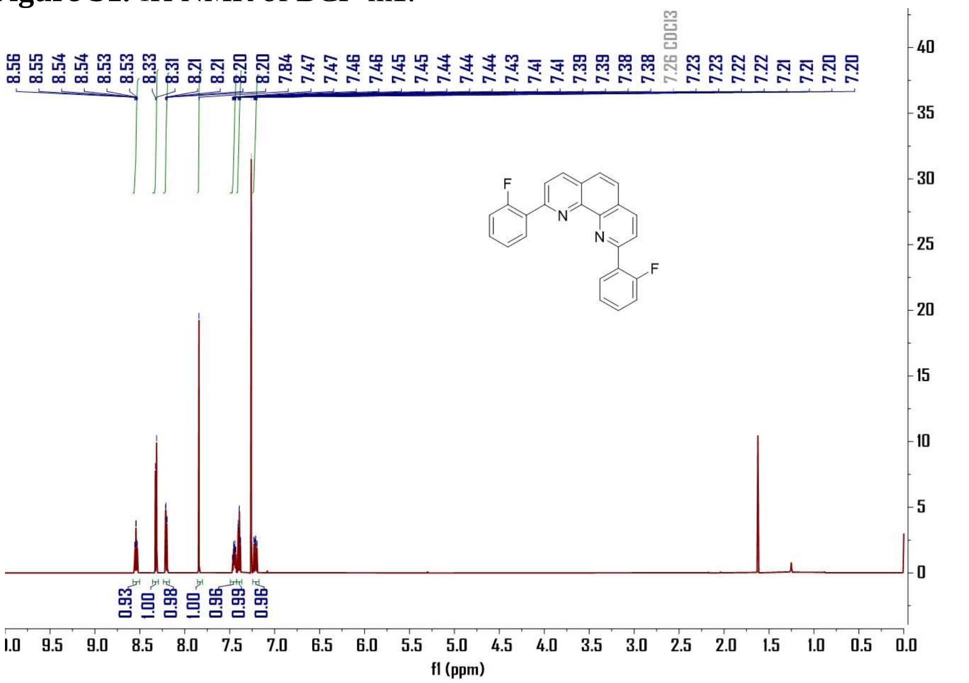

Figure S2. 1H NMR of BCP-m2F.

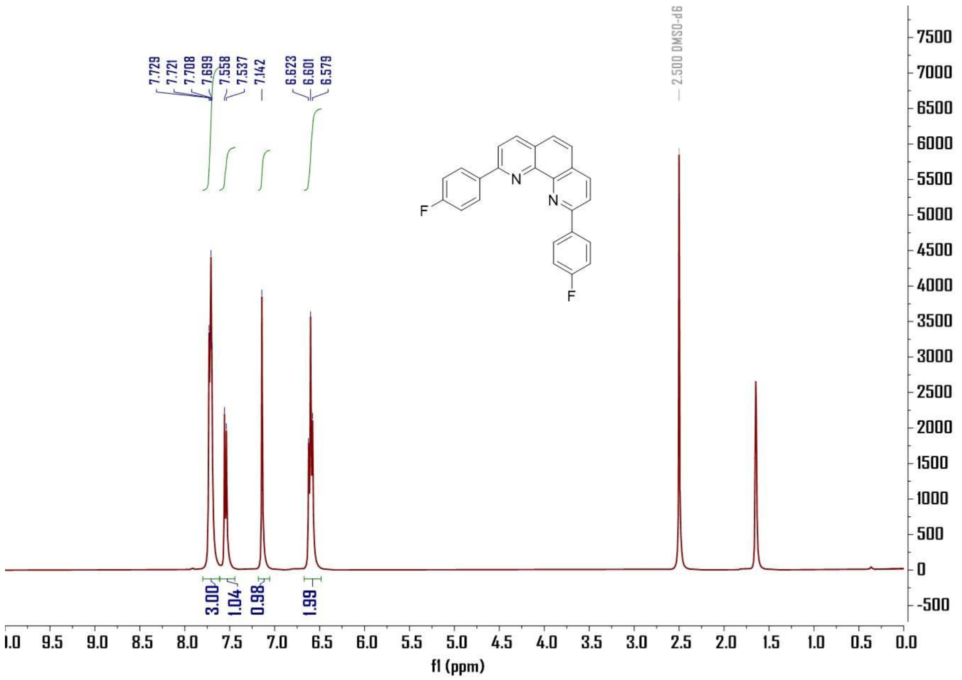

Figure S3. 1H NMR of BCP-m4F.

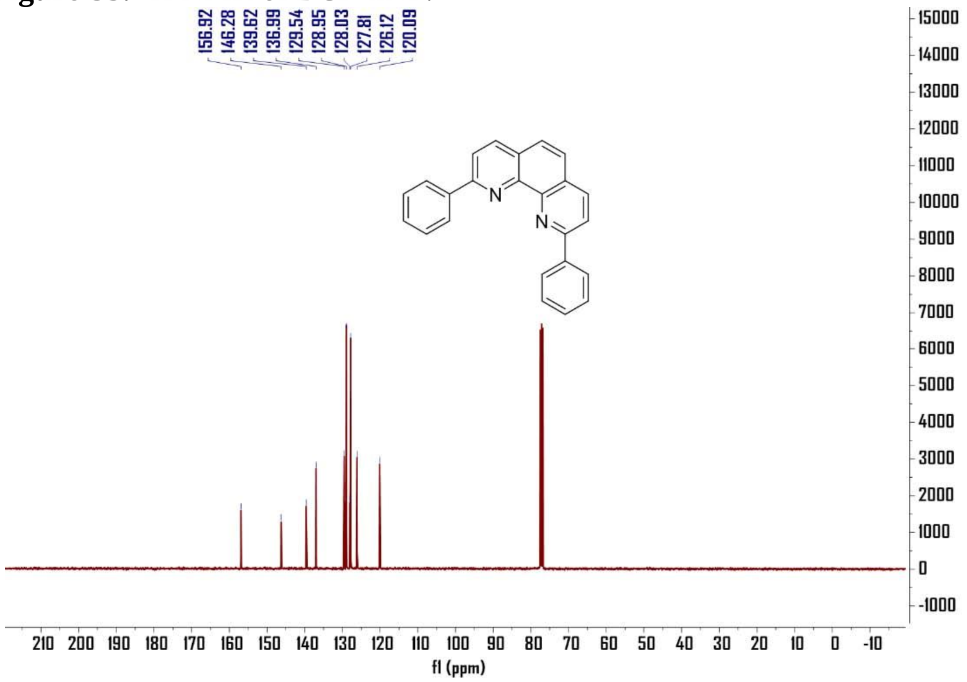

Figure S4. 13C NMR of BCP-m1.

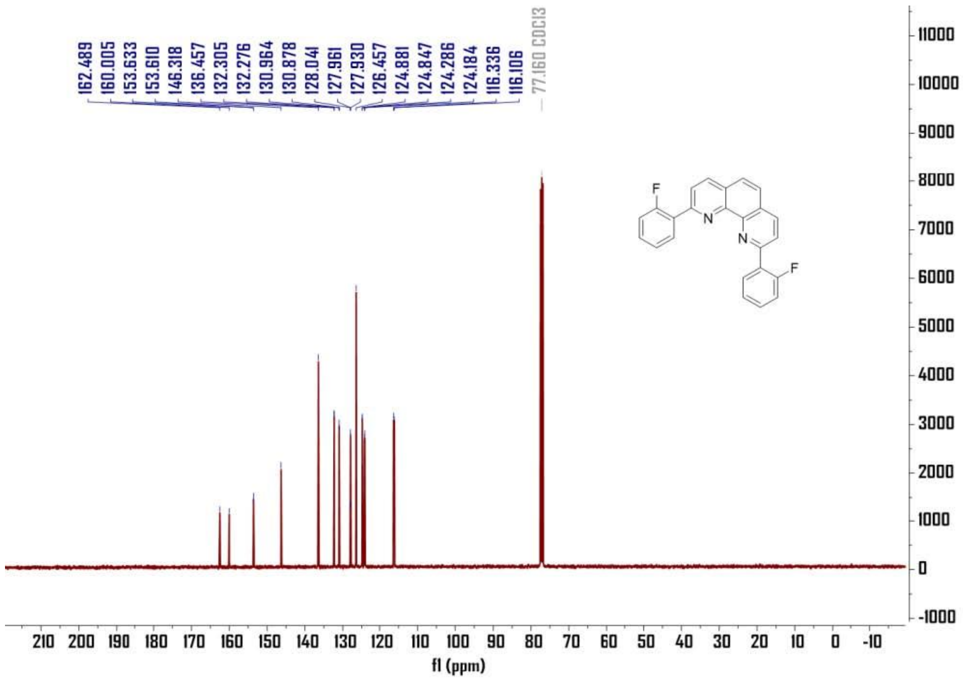

Figure S5. ^{13}C NMR of BCP-m2F.

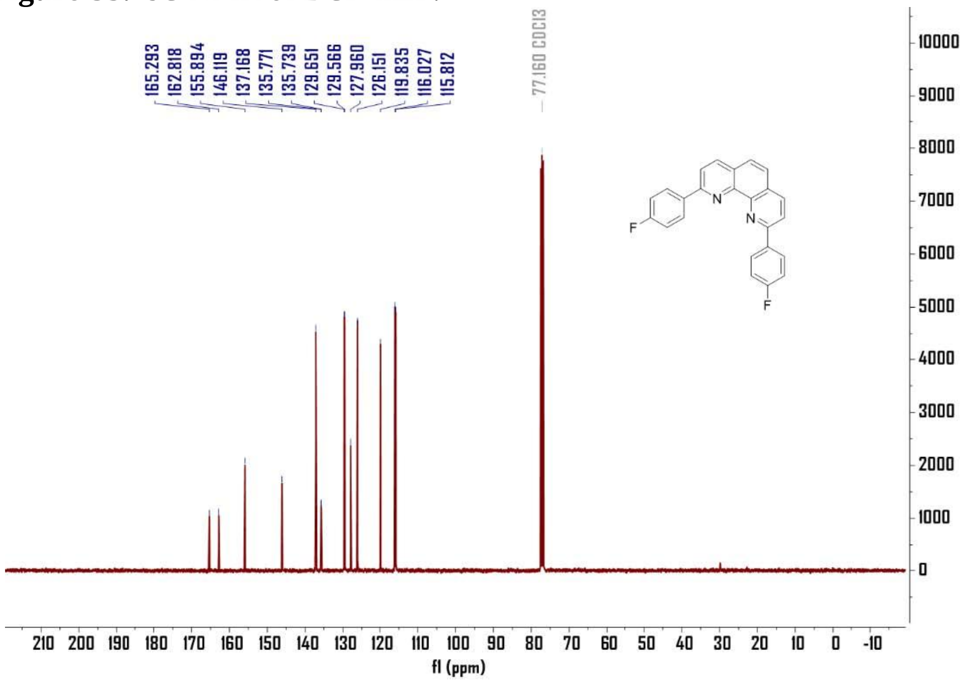

Figure S6. ^{13}C NMR of BCP-m4F

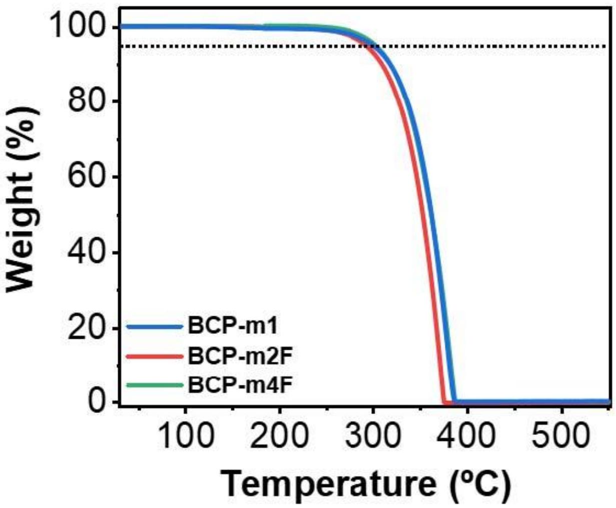

Figure S7. TGA of BCP-m1, BCP-m2F, and BCP-m4F.

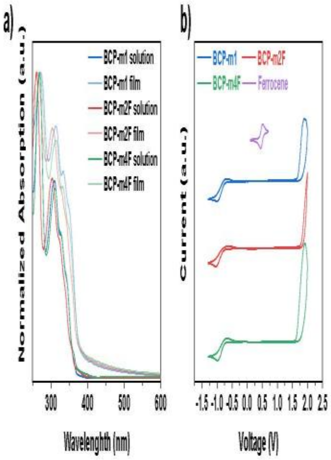

Figure S8. (a) Absorption spectra in solution and film state and (b) cyclic voltammograms of BCP, BCP-m1, BCP-m2F, and BCP-m4F.

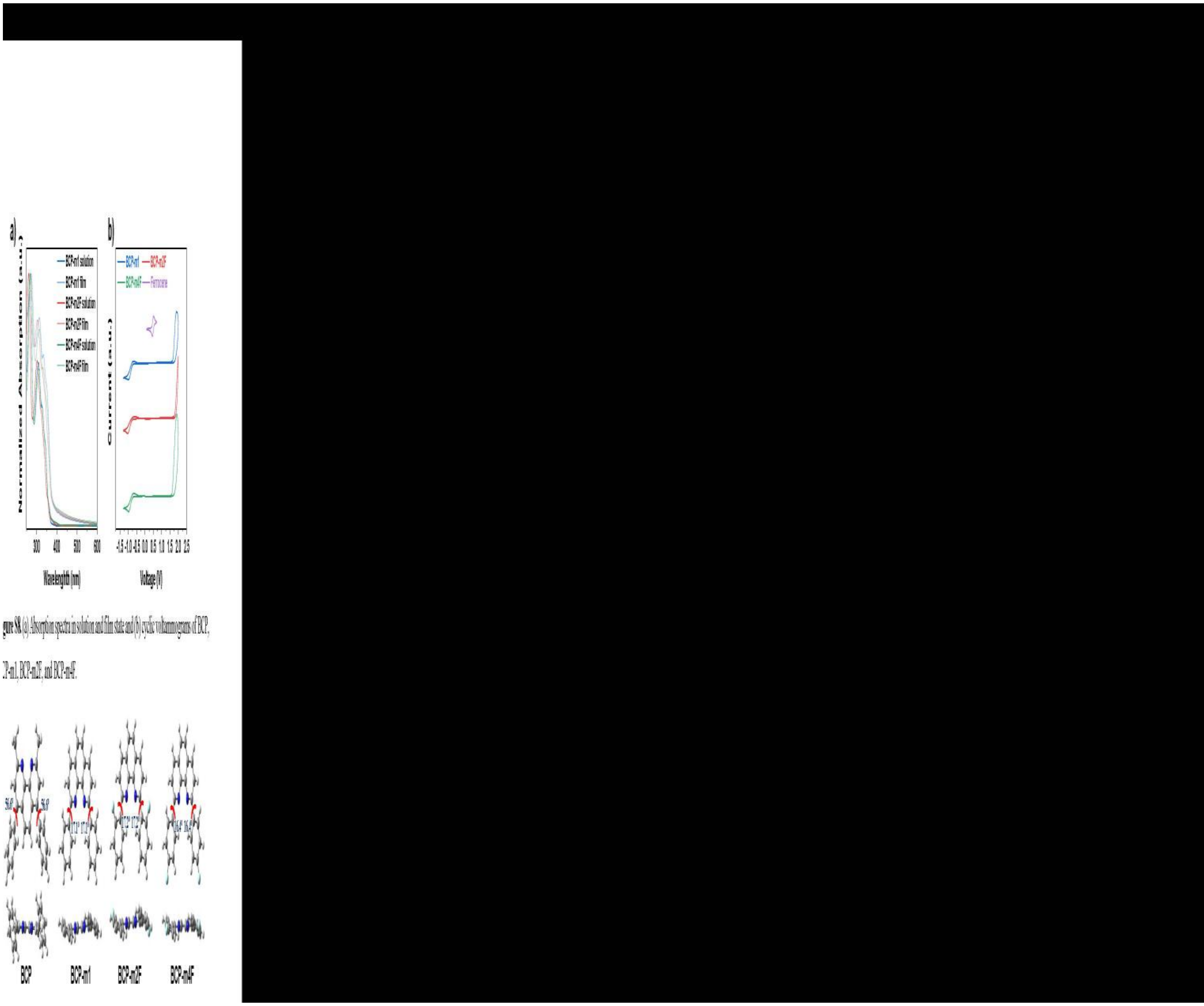

Figure S9. Molecular geometries of BCP, BCP-m1, BCP-m2F, and BCP-m4F in their neutral forms. Color-coded spheres represent hydrogen (white), carbon (gray), nitrogen (blue), and fluorine (cyan) atoms

[redacted]

Figure S10. Dipole moment of a) BCP-m1, b) BCP-m2F, and c) BCP-m4F. d) Electron reorganization energies of BCP-m1, BCP-m2F, and BCP-m4F.

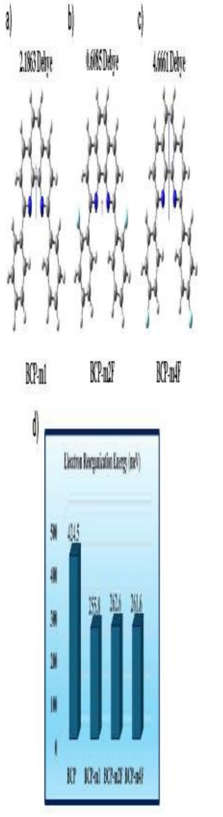

gure S10. Dipole moment of a) BCP-m1, b) BCP-m2F, and c) BCP-m4F. d) Electron rganization energies of BCP-m1, BCP-m2F, and BCP-m4F.

Figure S11. KPFM and EFM line profile of a) C_{60}, b) BCP-m1, c) BCP-m2F, and d) BCP-m4F.

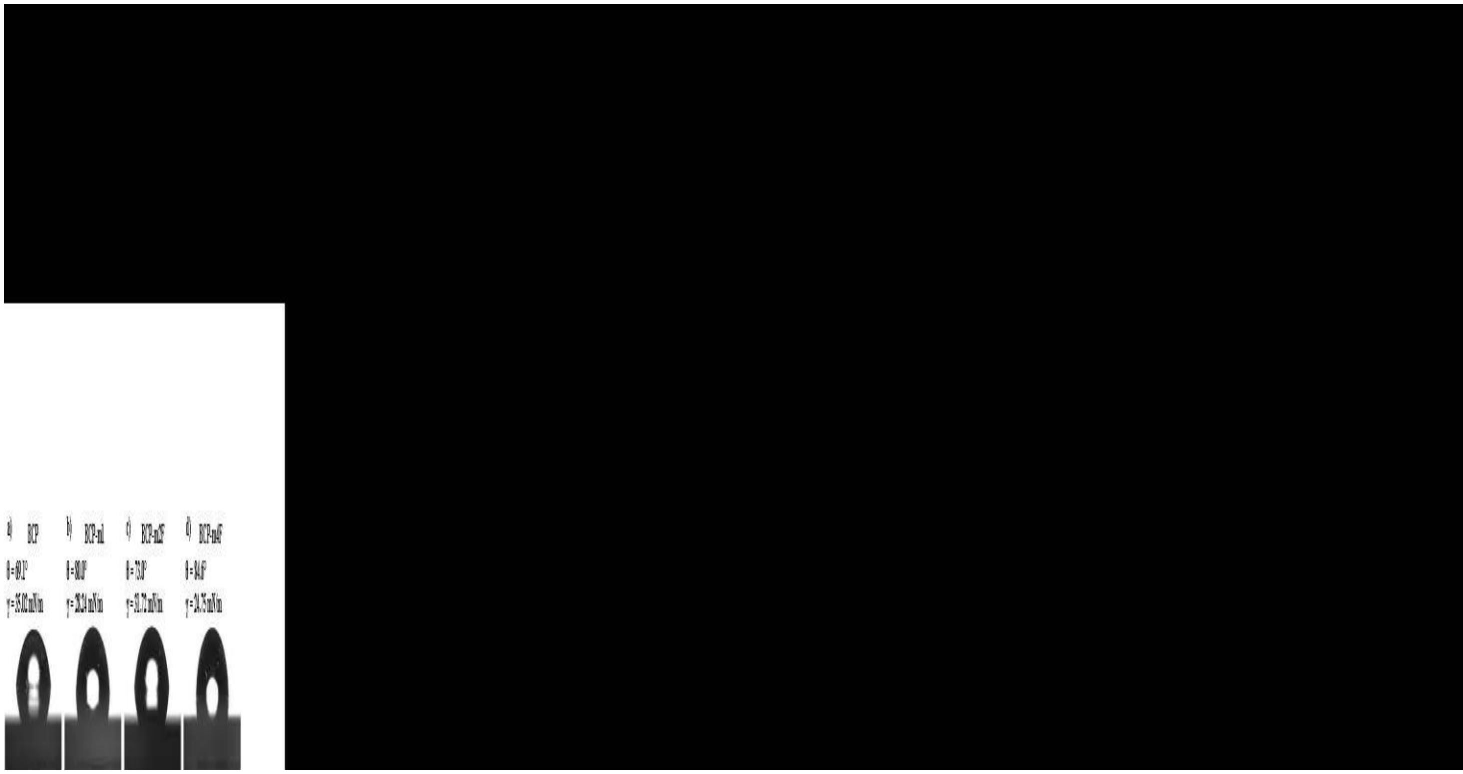

Figure S12. Water contact angle (θ) and surface energy (γ) of a) BCP, b) BCP-m1, c) BCP-m2F and d) BCP-m4F.

[redacted]

Figure S13. a,b) J-V characteristics, summary of c,d) V_{OC}-PCE and e,f) J_{SC}-FF of PSCs as a function of BCP-m2F and BCP-m4F thickness, respectively.

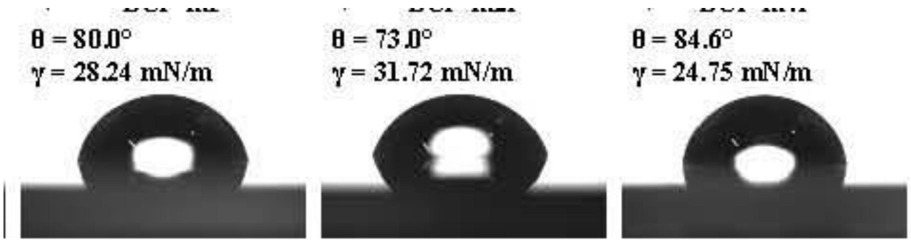

.ct angle (θ) and surface energy (γ) of a) BCP, b) BCP-m1, c) BCP-m2F

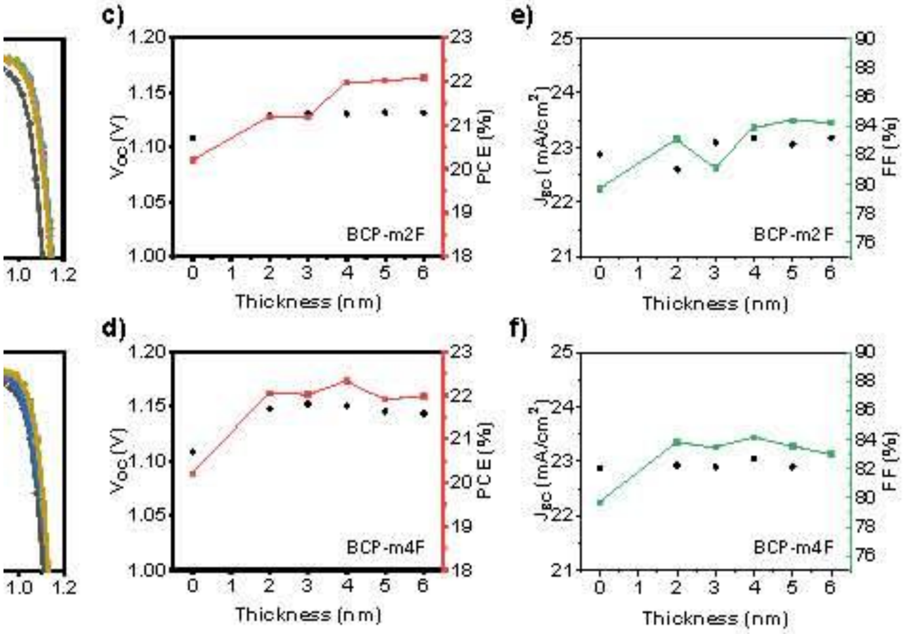

ıaracteristics, summary of c,d) V_{OC}-PCE and e,f) J_{SC}-FF of PSCs as a
d BCP-m4F thickness, respectively.

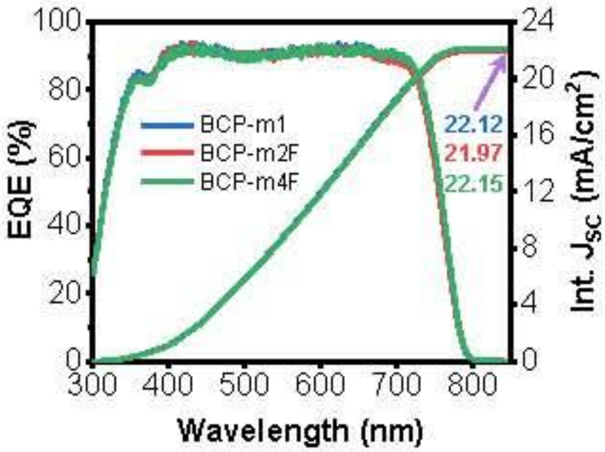

Figure S14. EQE spectra of PSCs using BCP-m1, BCP-m2F, and BCP-m4F

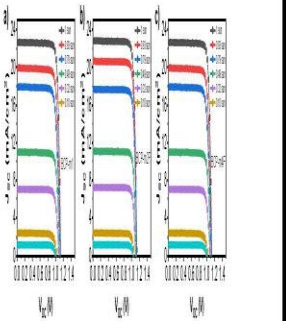

Figure S15. J-V characteristics of the PSCs using a) BCP-m1, b) BCP-m2F, and c) BCP-m4F as a function of light intensity.

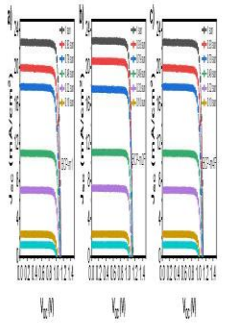

Figure S15. J-V characteristics of the PSCs using a) BCP-m1, b) BCP-m2F, and c) BCP-m4F as a function of light intensity.

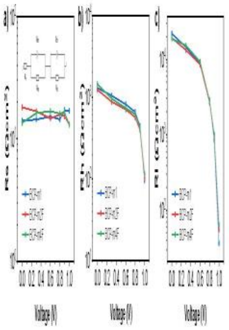

Figure S16. a) Series resistance, b) Impedance at high frequency, and c) Impedance at low frequency as a function of applied voltage. Under high frequency, chemical reactions could be measured.

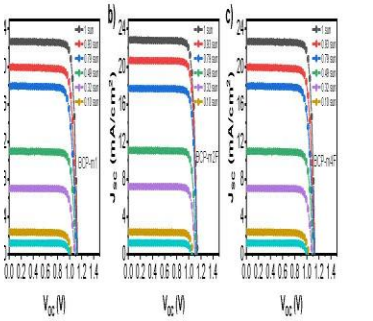

S15. J-V characteristics of the PSCs using a) BCP-m1, b) BCP-m2F, and c) BCP-m4F as
on of light intensity.

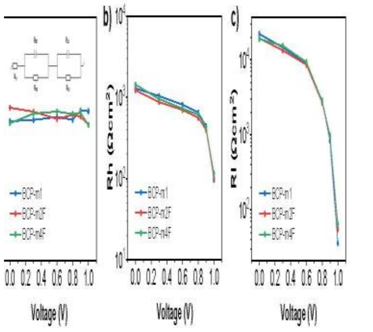

S16. a) Series resistance, b) Impedance at high frequency, and c) Impedance at low
cy as a function of applied voltage. Under high frequency, chemical reactions could be
ed.

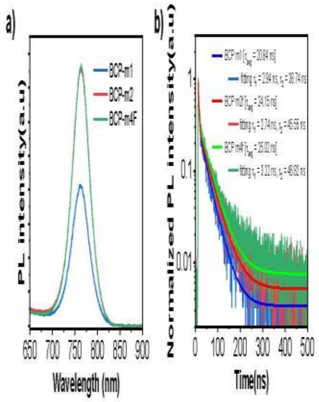

[redacted]

Figure S17. a) Steady state PL and b) TPRL of ITO/MeO-4PACz/Perovskite/C_{60}/BCP-m.

[redacted]

Figure S18. Detailed J-V characteristics from the damp heat stability test (ISOS-D3) of the PSCs using a) BCP, b) BCP-m1, c) BCP-m2F, and d) BCP-m4F.

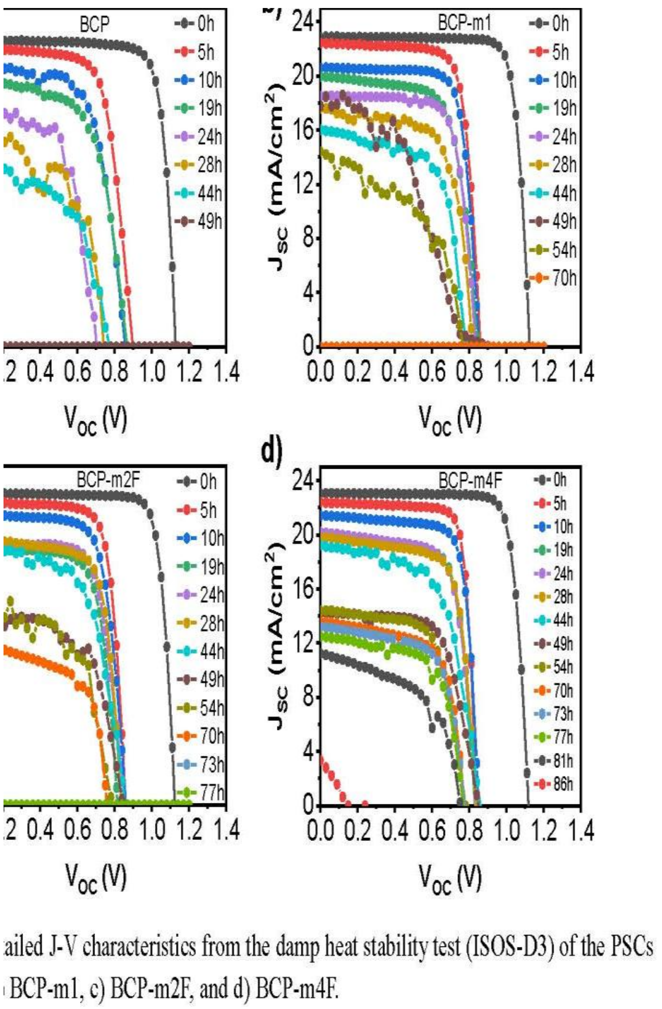

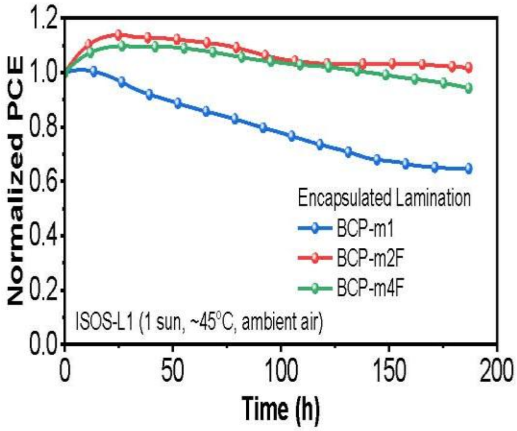

Figure S19. MPPT of the encapsulated PSCs using BCP-m1, BCP-m2F, and BCP-m4F.

Table S1. Extracted parameter obtained from absorption spectra and cyclic voltammograms of BCP-m1, BCP-m2F, and BCP-m4F.

**Materials**	**λ_{max} (nm)**	**λ_{onset} (nm)**	**E_{gopt} (eV)**		**E_{ox}/E_{HOMO} (V/eV)**	**E_{re}/E_{LUMO} (V/eV)**	**$E_{LUMOopt}$ (eV)**
					Solution	Film	Film
**BCP-m1**	268,309	272,314	376	3.30	1.69/-6.01	-0.77/-3.55	-2.71
**BCP-m2F**	260, 303	262, 303	374	3.32	1.81/-6.13	-0.79/-3.53	-2.81
**BCP-m4F**	268, 311	269, 313	376	3.30	1.64/-5.96	-0.78/-3.54	-2.66

Table S2. Photovoltaic performance parameters of PSCs as a function of BCP-m2F and BCP-m4F thickness.

	Thickness	JV J_{sc} [mA/cm2]	Voc [V]	FF [%]	Best PCE [%]
BCP-m2F	0 nm	22.87	1.108	79.69	20.20
	2 nm	22.60	1.128	83.07	21.19
	3 nm	23.09	1.131	81.10	21.18
	4 nm	23.18	1.131	83.87	21.98
	5 nm	23.06	1.132	84.37	22.02
	6 nm	23.18	1.131	84.21	22.09
BCP-m4F	0 nm	22.87	1.108	79.69	20.20
	2 nm	22.93	1.148	83.81	22.05
	3 nm	22.89	1.152	83.46	22.02
	4 nm	23.06	1.151	84.16	22.33
	5 nm	22.90	1.145	83.55	21.91
	6 nm	23.16	1.143	83.01	21.98

References

[1] M. J. Frisch, G. W. Trucks, H. B. Schlegel, G. E. Scuseria, M. A. Robb, J. R. Cheeseman, G. Scalmani, V. Barone, G. A. Petersson, H. Nakatsuji, X. Li, M. Caricato, A. V. Marenich, J. Bloino, B. G. Janesko, R. Gomperts, B. Mennucci, H. P. Hratchian, J. V. Ortiz, A. F. Izmaylov, J. L. Sonnenberg, D. Williams-Young, F. Ding, F. Lipparini, F. Egidi, J. Goings, B. Peng, A. Petrone, T. Henderson, D. Ranasinghe, et. al., Gaussian 16, Rev. B.01, Inc., Wallingford CT, 2016.

[2] A. D. Becke, *J. Chem. Phys.* **1993**, 98, 5648.

[3] A. D. Becke, *J. Chem. Phys.* **1996**, 104, 1040.

[4] C. Lee, W. Yang, R. G. Parr, *Phys. Rev. B* **1988**, 37, 785.

[5] GaussView, Version 6.1, Roy Dennington, Todd A. Keith, and John M. Millam, Semichem Inc., Shawnee Mission, KS, 2016.

[6] S. Grimme, J. Antony, S. Ehrlich and H. Krieg, *J. Chem. Phys.* **2010**, 132, 154104.

[7] S. Simon, M. Duran, and J. J. Dannenberg, *J. Chem. Phys.* **1996**, 105, 11024.
[8] H. Yi, D. Wang, L. Duan, F. Haque, C. Xu, Y. Zhang, G. Conibeer, A. Uddin, Electrochimica Acta. 319 (2019), 349-358
[9] I. Matacena, P. Guerriero, L. Lancellotti, B. Alfano, A. D. Maria, V. L. Ferrara, L. V. Mercaldo, M. L. Miglietta, T. Polichetti, G. Rametta, G. V. Sannino, P. D. Veneri, S. Daliento, Energies. 16 (2023), 13
[10] W. Clarke, G. Richardson, P. J. Cameron, Adv. Energy Mater. (2024) 2400955
